# Chicken or egg? Attribution hypothesis and nocebo hypothesis to explain somatization associated to perceived RF-EMF exposure

**DOI:** 10.3389/fpubh.2025.1561373

**Published:** 2025-04-09

**Authors:** S. Ariccio, E. Traini, L. Portengen, A. Martens, P. Slottje, R. Vermeulen, A. Huss

**Affiliations:** ^1^Institute for Risk Assessment Sciences, Utrecht University, Utrecht, Netherlands; ^2^PBL Environmental Assessment Agency, The Hague, Netherlands

**Keywords:** RF-EMF exposure, EMF, somatization, diagnosis, nocebo, attribution, perception

## Abstract

**Introduction:**

The aim of this study is to understand the temporal relationship between the somatization usually attributed to RF-EMFs, and to evaluate the attribution hypothesis and the nocebo hypothesis in this context.

**Method:**

In this longitudinal study, data from the Dutch Occupational and Environmental Health Cohort Study (AMIGO) was analyzed, consisting of a baseline questionnaire collected in 2011 (14,829 participants) and a follow-up questionnaire collected in 2015 (7,904 participants). Participants completed a questionnaire providing information on their health status, perceived environmental exposures, and demographics. Two sets of multiple regressions were conducted to evaluate the two hypotheses.

**Results:**

Results show that the attribution hypothesis overall explained symptom reporting in association to perceived RF-EMF base station exposure and perceived electricity exposure more frequently than the nocebo hypothesis.

**Discussion:**

This finding stands out from most of the existing literature, which primarily points to the nocebo effect as the main explanation for somatization in response to RF-EMF exposure. While this does not exclude, in absolute terms, the existence of a nocebo effect, potentially at other time scales, this finding has relevant consequences at the policy making level. The emerging relevance of the attribution hypothesis moves the focus on the discomfort of people with unexplained symptoms and their need to find a plausible explanation for their discomfort.

## Introduction

Since the introduction of wireless telecommunication technology, exposure to radiofrequency electromagnetic fields (RF-EMFs) has become essentially ubiquitous. Many people have reported experiencing symptoms that they attribute to the exposure. For instance, the presence of non-specific physical symptoms (NSPS) has been reported among people living in the vicinity of cell-phones base stations (1, 2) and people doing an extensive use of cell phones (3). Three hypotheses exist to explain this phenomenon: a biological effect, a nocebo effect, and an attribution effect. The biological hypothesis proposes that the symptoms are determined by biological processes related to RF-EMF exposure (4). Very limited to none scientific evidence exists to support this hypothesis (5, 6). For instance, a meta-analysis finds no evidence for a direct association between higher levels of RF-EMF exposure and frequency or severity of NSPS in either experimental or observational studies (7, 8).

If EMF-related symptoms are not of biological origin, then they are a case of somatization, i.e., the experience of somatic symptoms without an evident biological cause (9). Two hypotheses have been formulated that account for a somatization process of EMF perceived exposure. The nocebo hypothesis, instead, proposes that, since people expect RF-EMFs to cause somatization (typically due to media communication and alarmistic precautionary information), they will experience them just by feeling exposed to RF-EMFs, independently from objective exposure (4). In psychology, the experience of symptoms in association to an external cause, which is considered harmful even if it is actually innocuous, is named nocebo effect [e.g., (10)]. This has generally been considered the most plausible explanation for symptoms reported by people exposed to RF-EMFs [e.g., (10, 11)]. However, while there is clear evidence of immediate nocebo effects in experimental settings (12), there is uncertainty around longer-term nocebo effects.

Conversely, the attribution hypothesis proposes that attributing symptoms to RF-EMFs or other environmental exposures might be a way for people with long-lasting unexplained symptoms to find a putative cause of their poor health (4). The desire for definite knowledge is well-known in psychology and can bring people to draw closure by relying on incipient cues and limited information (34). This means that some people may feel more exposed to an environmental exposure, such as RF-EMFs, if they have unexplained symptoms they would like to find a cause of, to reduce uncertainty about their health issues (4).

This paper focuses on perceived, rather than measured, exposure, and therefore does not evaluate whether there is a biological effect. However, since it includes repeated measures of both perceived exposure and somatization at baseline (2011) as well at follow-up (2015), we were able to evaluate both the nocebo effect and attribution effect hypotheses. Given that the key difference between these hypotheses is the temporal relationship between somatization symptoms and perceived exposure, a longitudinal data collection was necessary to disentangle them. Thus, the AMIGO cohort, a Dutch cohort which has collected data on perceived exposure and health since 2011, was selected for this analysis which expands on previous similar approaches (13, 14).

## Aims

The aim of this study is to understand the temporal relationship between the somatization usually attributed to RF-EMFs and RF-EMF perceived exposure, and to evaluate the attribution hypothesis and the nocebo hypothesis in this context. The longitudinal design of this study is uniquely suited for this aim: the nocebo hypothesis posits that perceived exposure precedes somatization, while the attribution hypothesis posits that somatization precedes perceived exposure.

To investigate whether the observed relationships also apply to exposures with known health consequences, road exposure (noise and air pollution) and UV exposure were also considered. Road exposure, unlike RF-EMF exposure, is an exposure strongly associated with sensory perception (noise and smell). UV exposure, instead, is a natural RF-EMF exposure.

## Methods

### Study design and population

This study analyses data from the Dutch Occupational and Environmental Health Cohort Study (AMIGO) on environmental and occupational exposures, consisting of baseline questionnaire data collected in 2011 (14,829 participants) and follow-up questionnaire data collected in 2015 (7,904 participants). AMIGO was also included in LIFEWORK, a prospective cohort study on occupational and environmental risk factors and health in the Netherlands, which considered different environmental exposures, such as air pollution. See Slottje et al. (15) and Reedijk et al. (16) for extensive descriptions and profiles of these cohorts.

Participants completed a questionnaire providing, among other variables, information on their health status, perceived environmental exposures, and demographics. Age and sex were also included in the questionnaire. Age was asked as date of birth and then calculated as age in 2011. Sex was asked as a choice between male and female. The cohort included only participants at least 30 years old at baseline.

### Perceived exposure and environmental exposure

Perceived exposure to different environmental sources was initially measured with ten 7-step Likert items (from 0 = “not at all” to 6 = “very much”). See the Appendix for item wording.

Air pollution exposure was estimated as exposure to nitrogen dioxide (NO_2_). NO_2_ was measured between October 2008 and April 2011 during three 14-day periods to account for seasonal variation. The annual average NO_2_ concentrations were estimated at addresses of study participants at baseline using as predictor variables data on traffic intensity, household density, land use, and other study-area variables such as altitude and distance to the sea. A land-use regression model based upon annual average concentrations of NO_2_ was developed (see Beelen et al. (17) for a detailed description of the model development). No other objective exposure measure was included in the analyses.

### Health data

Somatization was measured with the Somatization scale of the Four-Dimensional Symptoms Questionnaire (4DSQ-S), ranging from 0 to 32 and used as a continuous variable in the analyses (10, 50).

Participants who stated that they had ever been diagnosed by a medical doctor with any of a predefined list of chronic conditions (e.g., asthma, osteoporosis, autism, type 2 diabetes) in either of the two waves of questionnaires were further classified as “diagnosed” (dichotomous variable). Participants who declared no diagnoses in either data collections were categorized as “not diagnosed”.

### Statistical analyses

Among the 7,905 participants who had answered the questionnaire both in 2011 and in 2015, 10.8% (*N* = 857) had missing data. Participants who skipped entire blocks of the questionnaire, not providing information on key variables, such as perceived exposure and somatization, were removed from the dataset (*N* = 638) For the remaining 219 participants with missing information, variables were imputed (median for ordinal and continuous variables, mode for categorical variables), before further statistical analyses. A total of 7,267 participants remained in our final analysis.

Distributions were investigated by computing the mean and standard deviation and using boxplots.

As part of the preliminary data elaboration, a principal axis exploratory factor analysis with Oblimin rotation (since factors were found to be correlated) was performed on the items of perceived exposure to define how many different dimensions of environmental exposure participants perceived (54). The analysis was run both at baseline and at follow-up to define a consistent factorial structure along the two time points and allow including factors from both years in the same analysis. Bartlett's K2(7) = 6,826.9, *p* < 0.001 for 2011 and Bartlett's K2(7) = 2,809.1, *p* < 0.001 indicated the list of perceived exposure items to be factorable. The number of factors to extract was based on Eigen values, scree test assessment, and parallel analysis. A threshold of 0.40 was considered for acceptable saturation scores (18). Mean scores were calculated for multi-item factors. Standardized Cronbach's alpha was then used to assess internal reliability, with acceptable reliability ranging between α = 0.70 and α = 0.95 (19).

**Figure 1:**
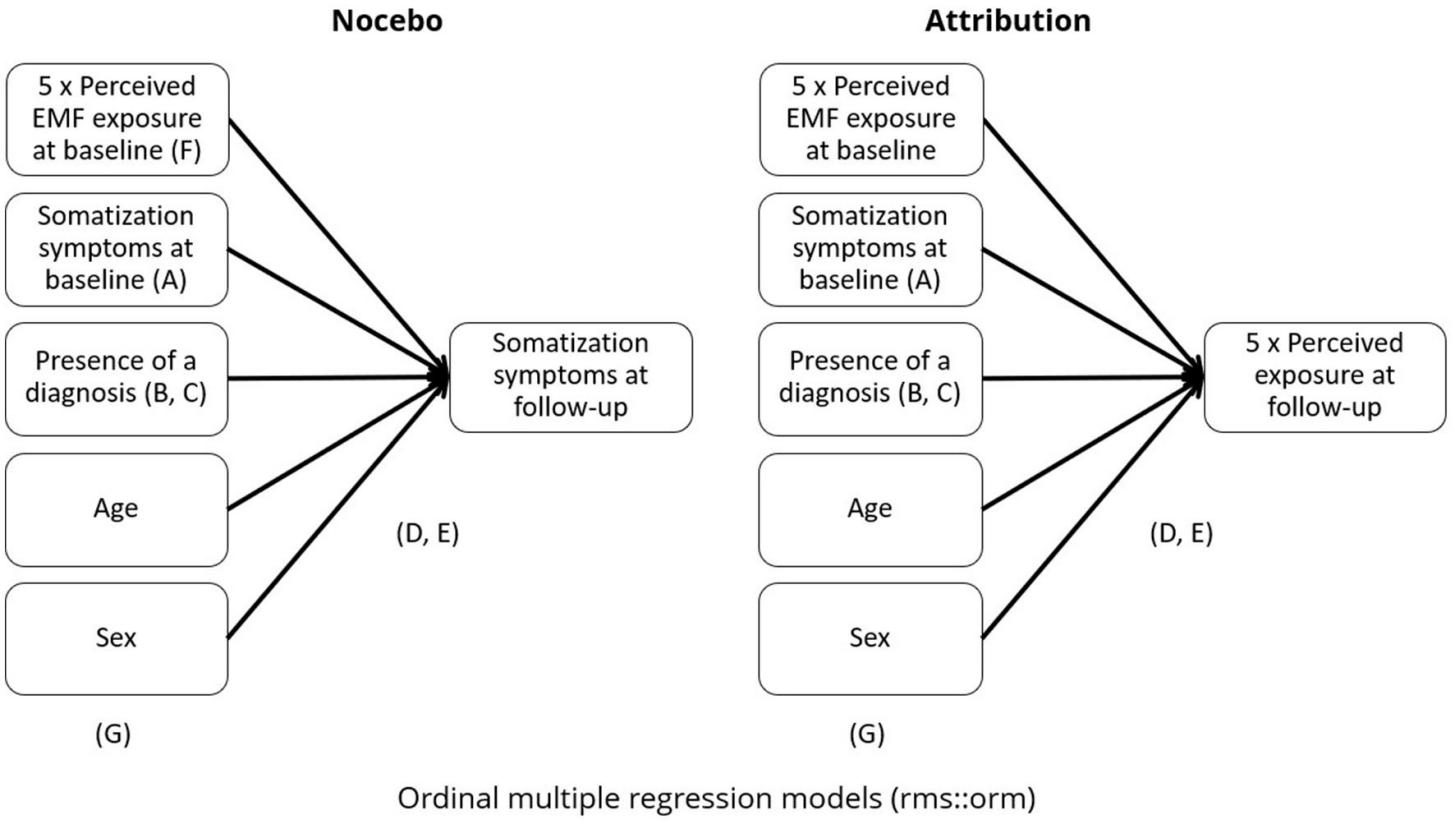
Summary of the main analyses. Letters indicate the focus of the different sensitivity and secondary analyses. A = without perceived exposure at baseline. B = without presence of diagnosis. C = restricted to diagnosed or not diagnosed participants. D = without adjustment for ordinal outcomes. E = non-linear regression. F = multi-exposure (only for Nocebo models). G= adjusted for NO_2_ exposure (only road exposure models).

Two sets of multivariable regression models were conducted as main analyses to evaluate the attribution hypothesis and the nocebo hypothesis. Figure 1 provides a graphical representation of the analyses and of the variables affected by the different sensitivity analyses.

The main analysis to evaluate the nocebo (NOC) hypothesis, consisted in estimating multivariable regression models with the somatization at follow-up (i.e., in 2015) as the dependent variable and the perceived exposure to the different exposure sources reported at baseline as the main predictor. The main analysis to evaluate the attribution hypothesis (ATTR), instead, consisted in multivariable regression models with the perceived exposure to the different exposure sources reported at follow-up (i.e., in 2015) as the dependent variable and the somatization at baseline as the main predictor. All analyses were adjusted for age and sex. The baseline scores of the dependent variables, i.e., perceived exposure for the attribution models and somatization for the nocebo models, were included in the main analyses to account for baseline differences. The presence or absence of a diagnosis by a medical doctor (at any time point) was also included in all main analyses since a diagnosis could provide an alternative cognitive explanation of the symptoms and it could thus work as a confounding variable.

**Figure 2:**
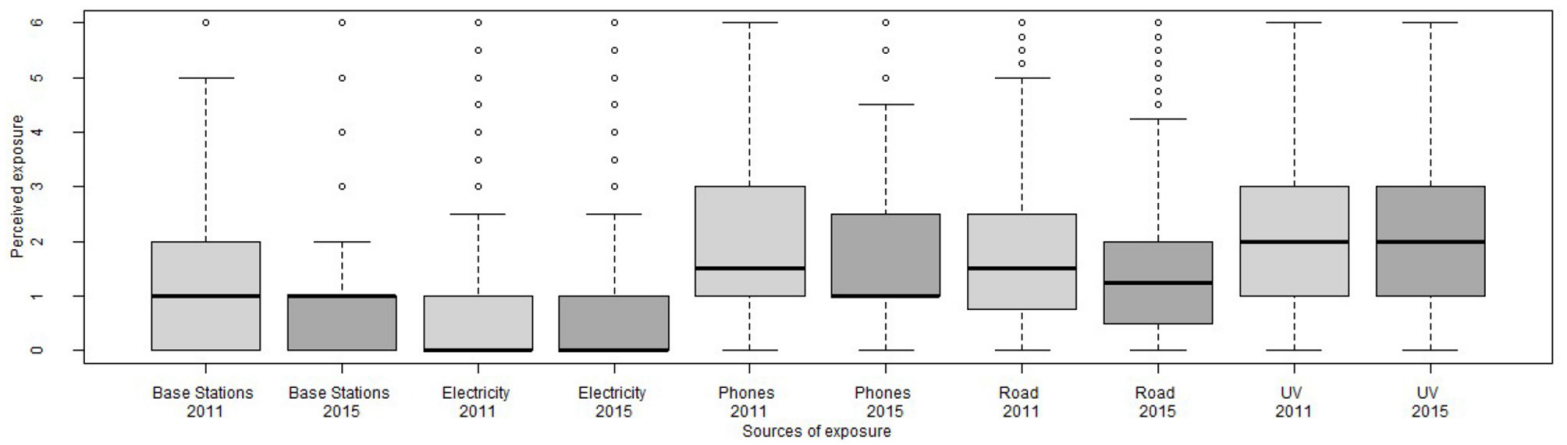
Distribution of perceived exposures at baseline (2011, light gray) and follow-up (2015, dark gray).

We performed a range of sensitivity analyses to estimate how far confounding or other factors could have affected our estimates: first, we repeated our main analyses (A) without baseline adjustment of the dependent variables, since baseline values of the outcome could also be considered as a potential confounding factor. Next, we removed the presence or absence of a diagnosis by a medical doctor from the baseline adjustment to check for the role of this variable (B). To further understand the role of the presence of a diagnosis, in further analyses (C) our models were stratified by participants who did or did not report a diagnosis (not diagnosed, *N* = 1,472; 20.3%) or at least one diagnosis (diagnosed, *N* = 5,792; 79.7%) at any time point. Since both perceived exposure and somatization were ordinal variables with a distribution that in most cases did not appear approximately normal, ordinal cumulative probability models for ordinal response variables with logistic distribution function were used as implemented in the orm function from R's RMS package (20). RMS is an *R* package focusing on providing several options for regression modeling, ranging from ordinal/logistic adjustment to non-linear modeling. We repeated analyses (D) also without adjustment for ordinal outcomes. Using the same package, another secondary analysis (E) tested the models for non-linearity, by running three-knot cubic splines. Wald tests were run, with RMS' ANOVA function, were used to test for the significance of the non-linear spline terms. Only for the nocebo hypothesis, an analysis (F) including all perceived exposures at baseline as predictors in a single regression (multi-exposure) model was also conducted. Finally, we explored the role of traffic related exposure in our model. We additionally adjusted our main road exposure models (both for attribution hypothesis and or nocebo hypothesis) for NO_2_ exposure (G). Figure 2 presents a summary of the models. All analyses were performed in R, version 4.4.1 (21).

## Results

### Descriptives

Preliminary analyses showed negligible differences between the whole sample answering to the baseline questionnaire (*N* = 14,829) and participants answering both to the first and to the second questionnaire (*N* = 7,264). Reported results thus focus only on participants for which both data points are available. Mean age of participants at baseline was 52.3 (SD= 8.98) years, 47% were men and 53% women; 56% participants had at best a secondary level education, while 41% had a tertiary education. Symptom scores ranged between 0 and 32. Eighty-two percent of the sample reported a score ranging between 0 and 10, i.e., “relatively normal physical tension symptoms”, 13% reported a score between 11 and 20, identified as “possible somatization symptoms”, and 2% reported a score higher than 20, labeled as “probable somatization symptoms”. In the present sample, the median reported score was 4. The most often reported somatization symptoms related to painful muscles and backpain (reported by more than 50% of participants) and neck pain (reported by more than 40% of participants). The most frequently reported symptoms at baseline were high blood pressure (more than 25% of the sample), high cholesterol (more than 15% of the sample), and arthrosis (more than 10% of the sample). Participants' NO_2_ exposure ranged from 12.07 μg/m^3^ to 57.96 μg/m^3^, with an average of 29.28 μg/m^3^.

The exploratory factor analysis (see Table 1) found a stable structure among the two time points composed of road traffic exposure (four items: air pollution from road traffic, air pollution from other sources, noise from road traffic, noise from other sources, α_2011_ = 0.84; α_2015_ = 0.81), electricity exposure (two items: EMF power lines, EMF transformer houses, α_2011_ = 0.78; α_2015_ = 0.79), and phone exposure (two items: EMF from mobile phones and EMF from cordless phones, α_2011_ = 0.78; α_2015_ = 0.83). Items “UV radiation sun” (from now on UV exposure) and “EMF mobile phone base station/radio/TV antennas” (from now on Base station exposure) were kept as single-item measures since they did not fit consistently within the factorial structure.

**Table 1:** Saturation scores of the exploratory factor analyses (Oblimin rotation) on items for measuring perceived exposure in 2011 and in 2015.

	**Baseline 2011 (63% of variance explained)**			**Follow-up 2015 (61% of variance explained)**		
	**Road perceived exposure (***α =* **0.84)**	**Electricity perceived exposure (**α = **0.78)**	**Phones perceived exposure (**α = **0.78)**	**Road perceived exposure (***α =* **0.79)**	**Electricity perceived exposure (**α = **0.79)**	**Phones perceived exposure (**α = **0.84)**
Air pollution road traffic	**0.84**	−0.05	0.05	**0.85**	−0.04	0.02
Air pollution other sources	**0.64**	0.16	−0.04	**0.64**	0.14	−0.05
Noise road traffic	**0.82**	−0.07	0.02	**0.77**	−0.05	0.03
Noise other sources	**0.67**	0.11	−0.05	**0.57**	0.10	−0.02
EMF mobile phones	0.11	0.10	**0.62**	0.04	0.01	**0.81**
EMF cordless phones	−0.02	−0.01	**0.93**	−0.03	0.00	**0.87**
EMF power lines	−0.02	**0.84**	0.02	−0.02	**0.80**	0.01
EMF transformer houses	0.04	**0.74**	0.02	0.02	**0.81**	0.02

Spearman's correlations between the different perceived exposure factors (including the one-item factors) ranged between ρ = 0.29 and ρ = 0.57 at baseline and between ρ = 0.28 and ρ = 0.56 at follow-up (see Table I in the Appendix). Bold values represent high saturation scores justifying the attribution of each item to a specific factor.

Figure 2 shows distributions for perceived exposures, while Table II (see Appendix) reports descriptive statistics for the main variables of this study. Reported perceived exposures were generally low, ranging (on a scale from 0 to 6) from 0.51 for perceived exposure to electricity at baseline to 2.08 for perceived UV exposure at follow-up.

### Nocebo hypothesis

As Table 2 shows, the multiple regressions testing the Nocebo hypothesis showed a positive, statistically significant, association between baseline perceived exposure and follow-up reported somatization for phone, road and UV exposure. Sensitivity analyses indicated that, for these exposures, effects were strongly driven by baseline somatization. Moreover, people without a diagnosis seemed to have overall a higher estimate than the people with a diagnosis. The higher estimates for the model without adjustment for ordinal variables suggests that the dependent variable might be approximate to a linear distribution. Wald tests indicated that the effect of the main predictor, i.e., baseline perceived exposure, was always substantially linear, since no non-linearity test was found to be significant. See Figure A in Appendix for a graphical representation of the main results.

**Table 2:** Estimates of multiple regression testing the nocebo hypothesis [95% confidence interval].

	**Base**	**Electricity**	**Phone**	**Road**	**UV**
NOC	0.00 [−0.03:0.04]	0.04 [−0.01:0.09]	0.05 [0.00:0.10]	0.10 [0.05:0.16]	0.07 [0.00:0.14]
NOC A	0.11 [0.08:0.15]	0.17 [0.12:0.22]	0.19 [0.14:0.24]	0.28 [0.23:0.33]	0.16 [0.09:0.23]
NOC B	0.00 [−0.03:0.04]	0.04 [−0.01:0.09]	0.05 [0.00:0.10]	0.10 [0.05:0.15]	0.08 [0.01:0.15]
NOC C—not diagnosed	0.10 [−0.06:0.25]	0.05 [−0.05:0.16]	0.14 [0.03:0.25]	0.26 [0.13:0.40]	0.24 [0.09:0.40]
NOC C—diagnosed	−0.01 [−0.05:0.03]	0.04 [−0.02:0.10]	0.03 [−0.03:0.09]	0.08 [0.02:0.13]	0.03 [−0.05:0.11]
NOC D	0.02 [−0.05:0.08]	0.11 [0.01:0.21]	0.08 [0.01:0.14]	0.13 [0.06:0.19]	0.05 [−0.02:0.12]
NOC F	−0.05 [−0.10:−0.01]	0.02 [−0.04:0.08]	0.04 [−0.03:0.10]	0.11 [0.05:0.18]	0.03 [−0.05:0.10]
NOC G	-	-	-	0.11 [0.05–0.16]	-

Dependent variable: somatization at follow-up. NOC: ordinal model including perceived exposure, and presence of diagnosis, adjusted for age, sex, and somatization at baseline. NOC A, same as NOC, without somatization at baseline. NOC B, same as NOC, without presence of diagnosis. NOC C without diagnosis: same as NOC B, restricted to participants not diagnosed. NOC C with diagnosis: same as NOC B, restricted to participants diagnosed. NOC D, same as NOC, but without adjustment for ordinal outcomes. NOC F, same as NOC, but mutually controlling for other exposures. NOC G road exposure model adjusted for NO_2_ modeled exposure at baseline. Note that results of NOC E (spline analysis) can be found in Table III, in the Appendix.

### Attribution hypothesis

As shown in Table 3, results indicate positive, and statistically significant, associations between baseline reported somatization and follow-up perceived exposure for base station exposure, electricity exposure, and road exposure. Sensitivity analyses showed that effects were consistent both when removing the presence of diagnosis from the analysis and when not controlling for perceived exposure at baseline.

**Table 3:** Estimates of multiple regressions testing the attribution hypothesis [95% confidence interval].

	**Base**	**Electricity**	**Phone**	**Road**	**UV**
ATTR	0.11 [0.05:0.16]	0.12 [0.06:0.17]	0.03 [−0.02:0.08]	0.09 [0.04:0.14]	0.04 [−0.01:0.09]
ATTR A	0.20 [0.15:0.25]	0.21 [0.15:0.27]	0.12 [0.07:0.17]	0.25 [0.20:0.31]	0.07 [0.02:0.13]
ATTR B	0.10 [0.05:0.15]	0.11 [0.05:0.17]	0.04 [−0.01:0.09]	0.09 [0.04:0.14]	0.05 [0.00:0.1]
ATTR C—not diagnosed	0.06 [−0.05:0.18]	−0.02 [−0.14:0.11]	0.05 [−0.06:0.16]	0.05 [−0.05:0.16]	−0.06 [−0.17:0.05]
ATTR C—diagnosed	0.13 [0.06:0.19]	0.15 [0.08:0.22]	0.03 [−0.03:0.09]	0.10 [0.04:0.16]	0.06 [0.00:0.13]
ATTR D	0.01 [0.01:0.02]	0.01 [0.00:0.01]	0.00 [0.00:0.01]	0.01 [0.00:0.01]	0.01 [0.00:0.01]
ATTR G	-	-	-	0.09 [0.04–0.14]	

Dependent variable: perceived exposure at follow-up. ATTR, ordinal model including perceived exposure, and presence of diagnosis, adjusted for age, sex, and perceived exposure at baseline. ATTR A, same as ATTR, without perceived exposure at baseline. ATTR B, same as ATTR, without adjustment for presence of diagnosis. ATTR C not diagnosed: same as ATTR B, restricted to participants without diagnosis. ATTR C diagnosed: same as ATTR B, restricted to participants with a diagnosis. ATTR D, same as ATTR, but without adjustment for ordinal outcomes. ATTR G road exposure model adjusted for modeled NO_2_ exposure at baseline. Note that there was no ATTR F, and that results of ATTR E (spline analysis) can be found in Table III, in the Appendix.

Stratified analysis showed that the effect was only significant in the diagnosed sample and not in the not diagnosed sample. This could be possibly due to the smaller sample size of the not diagnosed sub-sample (*N* = 1,472), vs. the diagnosed sample (*N* = 5,792). Wald tests indicated that the effect of the main predictor, i.e., baseline reported somatization, was always substantially linear, since no non-linearity test was found to be significant. See Figure B in the Appendix for a graphical representation of the main results.

## Discussion

### Summary of results

The aim of the current study was to evaluate the attribution hypothesis and the nocebo hypothesis that have been formulated to explain non-specific physical symptoms people experience, in association with exposure to RF-EMFs. The association between RF-EMF exposure (from mobile phone base stations, and electricity) and somatization was found consistently in models evaluating the attribution hypothesis and not in models based on the nocebo hypothesis. UV exposure was only found in the models assessing the nocebo hypothesis, while road exposure was associated to somatization in models of both kinds. Phone exposure was not consistently associated to somatization in our study population.

According to these results, the attribution hypothesis overall explained symptom reporting in association to perceived RF-EMF base station exposure and perceived electricity exposure more frequently and to a stronger extent than the nocebo hypothesis. This finding stands out from most of the existing literature, which primarily points to the nocebo effect as the main explanation for somatization in response to RF-EMF exposure.

The tendency of people to rely on external sources of control when they are not in direct control of events has been proposed and developed by several psychological theories and perspectives (22). Previous research has already shown that lacking control makes people more likely to perceive a variety of illusory patterns, including seeing images in noise, forming illusory correlations, and developing superstitions (23). This is consistent with the attribution hypothesis: attributing symptoms to RF-EMF exposure might be a way for people with long-lasting unexplained symptoms to find an explanation to their poor health. In this sense, attributing symptoms to RF-EMF might be a way for people with unexplained symptoms to find a cognitive closure and thus solve the discomfort of not knowing the cause of their symptoms.

Receiving a diagnosis can affect the implicit and explicit expectations patients have about the course of the disease and it can ultimately lead to changes in the disease progression and in symptom reporting [see Illness Expectation by Pagnini (24)]. Receiving a diagnosis, when reported symptoms are otherwise not visible and hard to prove, is considered as a relief and as a way to be socially recognized as a sick person, with related psychological and practical benefits (25). One of these positive psychological effects is increasing personal control (26). Since receiving a chronic diagnosis by a doctor might give patients a plausible explanation for their symptoms [e.g., (26)], the effect of diagnosis was also considered in our analysis. While a confounding effect of the presence of a diagnosis does not emerge from the sensitivity analysis; the stratified analysis indicates that it could have an interaction effect. Splitting the sample between diagnosed and non-diagnosed participants indicated that participants without diagnosis (vs. with diagnosis) had stronger nocebo effect and weaker attribution effect. This could suggest that, unlike as what has been suggested elsewhere, receiving a diagnosis might lead to reporting fewer symptoms after feeling exposed (nocebo effect) and yet feeling in general more exposed to external noxious sources (attribution effect).

The present results do not exclude that the nocebo effect might play a role in people's experience of RF-EMF exposure: the presented study was limited to two, relatively distant, time points (2011 and 2015). Most studies finding nocebo effects in this context, instead, have focused on experimental designs in which participants are usually requested to report their symptoms within a few minutes or hours of their real of sham RF-EMF exposure. It is possible that, once people have made the cognitive attribution of symptoms to RF-EMF exposure, they might experience a nocebo effect in specific moments, i.e., if they perceive to be exposed to RF-EMFs, then they experience those symptoms [see also Common-Sense Model of Illness Representation, Diefenbach and Leventhal, (53)]. As a consequence, nocebo and attribution hypotheses might both apply to RF-EMF experience, but with different timeframes or time windows. It is also possible that one of the two hypotheses is more adequate for specific people, for instance depending on the sources of information they have and how much they trust the information they receive, or if they have unexplained symptoms or not.

### Strengths and limitations

This study's main strengths are the longitudinal design, with repeated information on a big sample about both perceived exposure to a range of environmental factors and somatization, thus allowing to test for confounding effects of baseline measurements and the inclusion of several other potential confounding factors. This study is among the first to provide longitudinal evidence of the attribution hypothesis in the context of environmental exposures including RF-EMF exposure [see also Martens et al. (14, 27)]. However, our result should be interpreted with a number of caveats and contextual considerations in mind.

A significant limitation of our study is that, besides for road exposure, it did not include objective measures of exposure, so it is impossible to disentangle perceived exposure from real exposure. In particular, the study does not include objective measurements of RF-EMF exposure. However, nocebo and attribution are two eminently psychological phenomena that, especially for an exposure that is not sensorily perceived, such as RF-EMFs [see Bosch-Capblanch et al. (7)], are likely to be independent from objectively measured exposure, as previously shown by Martens and colleagues on this same cohort, focusing on estimated downlink RF-EMF exposure (13, 14). It is worth mentioning how the pattern of results that emerged was different for road and UV exposure, i.e., exposures associated to sensory stimulations (i.e., noise and smell), and UV radiation exposure that is an electromagnetic field, but not technology-related and is usually perceived with less alarm. As for what concerns road exposure, since the items composing this indicator make reference to exposures associated to sensory cues such as noise and smell, a speculative hypothesis is that people reporting high perceived road exposure are those living in environments where this exposure sources are indeed relatively high (e.g., urban environments). This would thus be an accurate report of these exposures, which are from the literature known to be associated with health issues that might be reported here as somatization (28), thus suggesting a biological noxious effect of road exposure. Similarly, the positive associations found between baseline UV exposure and follow-up somatization might indicate a noxious biological effect of objective UV exposure, in line with existing literature. However, the negligible effect of including NO_2_ exposure in the models reinforces the idea that perceived and real exposure are substantially unrelated. For instance, in the nocebo model, the estimate for a NO_2_ exposure effect on reported symptoms was −0.02 [−0.07:0.04] and including NO_2_ exposure did not affect estimates in the related model.

It should be noted that several marginal results were found, i.e., many regression scores were very close to 0. On the one hand this is an indication of phenomena that overall have small effects, and where many other variables are at play. Moreover, it could be due to non-optimal measuring and scaling of the variables, which is a common issue when dealing with psychological variables such as perceived exposure and somatization. The sensitivity analyses help interpreting these results, especially showing which ones are more stable and consistent, such as those related to base stations and road exposure and which are more ambiguous, such as those concerning phone exposure.

Another potential limitation of the present study is the operationalization of the variable presence of a diagnosis. In this study, this was considered as a dichotomic variable, focusing on the presence/absence of any diagnosis. Treating diagnosis as a binary variable limits the depth of the analysis. This operationalization was chosen because the focus of the study was on somatization, that is known to be unspecific and potentially associated with a variety of potential diagnoses. However, each diagnosis may be a plausible explanation for some, but not all, symptoms a person experiences. Future studies differentiating by diagnosis type could provide more nuanced insights into how different medical conditions influence symptom attribution.

### Further research and policy implications

This study focused on a population sample from the general Dutch population and did not take specific psychological traits into account. Future studies should try to investigate the attribution hypothesis and the nocebo hypothesis in relation to RF-EMF exposure among specific populations, such as people self-defining as RF-EMF hypersensitive and/or people reporting non-typical levels of somatization and unexplained symptoms (see already Martens et al. (14, 27) for some work in this direction). Moreover, while this study did not include them, psychological traits and states such as self-confidence, neuroticism, anxiety, and optimism, which have already been studied in relation to the exposure- related nocebo effect [e.g., (29, 30)] are likely to also affect the attribution effects and, potentially, moderate both phenomena, for instance, traits such as anxiety or health beliefs may influence symptom attribution (30–32). Variables such as risk perception, knowledge about the different exposure sources, and previous experience should also be considered, consistently with the Social Amplification of Risk Framework (33). In this sense, the lack of consistent relationship between somatization symptoms and perceived phone exposure suggests that other psychological phenomena could play a role. Lastly, while the nocebo effect has already been deeply investigated in psychology, the relationship of the attribution effect with existing psychological paradigms such as need of cognitive closure (34), the compensatory control theory (35), and cognitive dissonance (36) should be further investigated.

Further studies should look into the role of the presence of a diagnosis more closely and try to disentangle among participants who have received a diagnosis, those who have received a diagnosis that explains all their reported symptoms and those who have received a diagnosis but still have unexplained symptoms. It is possible that these different groups will have different tendencies to experience an attribution effect. In this sense it should be noted that receiving a diagnosis [especially stage-based diagnoses, Rutjens et al. (52)] can be a way for patients to regain control over their own story, health and wellbeing. Thus, if the diagnosis is lacking, it is plausible that patients search for another source of control to explain their discomfort. Attributing a negative influence to external entities, e.g., environmental exposures, could compensate for threats to control and thus enhances wellbeing, even if that entails having an “enemy” (37, 38). More qualitative studies, such as those already conducted by Dieudonné (39), would also provide richer insights into the psychological process behind symptom attribution. As for what concerns perceived exposure, because of the lack of validated measures of perceived exposure to environmental sources, the present composite measures have been issued from an exploratory factor analysis. Participants in our study associated perceived exposure depending on their life-contexts more than on their objective features, i.e., associating noise and air pollution in the road factor, but not antennas, phone, and electricity. Similar analyses should be conducted in other contexts, since understanding how people associate the different perceived exposures in different contexts could inform risk communication policies. For instance, in the case of local installations of new power lines, miscommunication between residents and the responsible parties for the project (companies and local authorities involved), and consequent negative expectations and perceived injustice, were found to be associated with negative experiences and poor acceptance of new high-voltage power line projects (40). However, some studies found instead that more than the precautionary information, predictors of perceived symptoms was prior to risk perception, mediated by symptom expectations [e.g., (41)]. For instance, a Dutch field study found a positive association between proximity to a new high-voltage power line and symptom reports, and the belief that these reported symptoms were caused by a power line. The belief that a power line could cause these symptoms was at baseline already stronger for residents living close compared to residents living farther away (42).

Public health interventions should thus focus on providing correct and actionable information about RF-EMF, especially since research shows that not every kind of information has the same effect on EMF risk perception and acceptance. Sharing measurements of personal EMF exposure seems to not affect significantly risk perception [(43), but see Ramirez-Vazquez et al. (44)], but it improves confidence in individual's ability to self-protection (45, 46). Explaining the distance–exposure relationship in EMF systems is reported to accurately lower risk perception (51) and providing information about how to reduce exposure seems to be more efficient in increasing EMF acceptance than providing technical information about this technology (47) and does not seem to trigger nocebo responses (41). As for what concerns communication of results of epidemiological research on EMF, Freudenstein et al. find that, for an effective risk communication and avoiding unnecessarily increase risk perception, it is important to clearly differentiate between risk assessment and hazard identification and to report study outcomes in full, rather than selectively, e.g., when presenting associations between EMF and specific health outcomes (48, 49).

### Chicken or egg?

Overall, by comparing the nocebo effect and the attribution effect hypotheses within the same longitudinal dataset, this study provides some new perspective on the relationship between somatization and perceived exposure, suggesting that symptom reporting in association to perceived RF-EMF exposure is better explained by the attribution hypothesis than by the nocebo hypothesis, at least at the time scale considered by this study. While this does not exclude, in absolute terms, the existence of a nocebo effect, potentially at other time scales, this finding has relevant consequences at the policy making level.

While the focus on the nocebo hypothesis, still prevalent in literature, highlights the role of the media in diffusing inaccurate perceptions about new sources of exposure, such as RF-EMFs; the emerging relevance of the attribution hypothesis moves the focus on the discomfort of people with unexplained symptoms and their need to find a plausible explanation for their discomfort and thus to the importance of receiving a diagnosis. The two hypotheses do not necessarily exclude each other, since it is possible that people will start to experience nocebo effects of exposures once they have attributed their symptoms to them. However, policy makers should devote more attention to take into account the discomfort of patients with long-lasting unexplained symptoms, providing psychological support and, hopefully contributing to the definition of a diagnosis, which is known to contribute to patients' wellbeing by providing an explanation and an identity. However, the role of diagnosis should be further investigated in the future.

## Data Availability

The data analyzed in this study is subject to the following licenses/restrictions: Data can be shared after establishing a formal collaboration. Requests to access these datasets should be directed to amigoproject@uu.nl.
