# Supplementary material for: Chicken or egg? Attribution hypothesis and nocebo hypothesis to explain somatization associated to perceived RF-EMF exposure
Source: Front Public Health. 2025 Apr 9;13:1561373. doi: 10.3389/fpubh.2025.1561373 (PMC12014461; doi:10.3389/fpubh.2025.1561373)
Supplement: Supplementary file 1 [file Supplementary_file_1.docx]

**APPENDIX**

Items to measure perceived exposure

- To what extent do you think you are exposed to air pollution by road traffic?
- To what extent do you think you are exposed to air pollution by other sources?
- To what extent do you think you are exposed to noise by road traffic?
- To what extent do you think you are exposed to noise by other sources?
- To what extent do you think you are exposed to UV radiation of the sun?
- To what extent do you think you are exposed to EMF of mobile phone basestation/Radio/TV antennas?
- To what extent do you think you are exposed to EMF of mobile phones?
- To what extent do you think you are exposed to EMF of cordless phones?
- To what extent do you think you are exposed to EMF of power lines?
- To what extent do you think you are exposed to EMF of transformer houses?

Items to measure the presence of diagnosis:

- Have you ever been diagnosed by a doctor with asthma ?
- Have you ever been diagnosed by a doctor with COPD?
- Have you ever been diagnosed by a doctor with cancer?
- Have you ever been diagnosed by a doctor with angina?
- Have you ever been diagnosed by a doctor with heart attack (MI)?
- Have you ever been diagnosed by a doctor with other heart conditions?
- Have you ever been diagnosed by a doctor with stroke/transient ischaemic attack?
- Have you ever been diagnosed by a doctor with high blood pressure?
- Have you ever been diagnosed by a doctor with artrosis?
- Have you ever been diagnosed by a doctor with arthritis/rheumatoid arthritis?
- Have you ever been diagnosed by a doctor with chronic back disorder/hernia?
- Have you ever been diagnosed by a doctor with Type 1 diabetes (“insulin-dependent” or juvenile diabetes)?
- Have you ever been diagnosed by a doctor with Type 2 diabetes (“non-insulin-dependent” or late-onset diabetes)?
- Have you ever been diagnosed by a doctor with unknown type of diabetes?
- Have you ever been diagnosed by a doctor with high cholesterol?
- Have you ever been diagnosed by a doctor with chronic liver disease?
- Have you ever been diagnosed by a doctor with thyroid-related disorders (Increased or decreased metabolism)?
- Have you ever been diagnosed by a doctor with inflammory bowel syndrome?
- Have you ever been diagnosed by a doctor with stomach ulcer?
- Have you ever been diagnosed by a doctor with chronic fatigue syndrome?
- Have you ever been diagnosed by a doctor with depression?
- Have you ever been diagnosed by a doctor with ADHD?
- Have you ever been diagnosed by a doctor with deafness/partial hearing loss?
- Have you ever been diagnosed by a doctor with cataract?
- Have you ever been diagnosed by a doctor with glaucoma or high eye pressure?
- Have you ever been diagnosed by a doctor with migraine?
- Have you ever been diagnosed by a doctor with concussion?
- Have you ever been diagnosed by a doctor with epilepsy?
- Have you ever been diagnosed by a doctor with Parkinson’s disease?
- Have you ever been diagnosed by a doctor with Amyotrophic Lateral Sclerosis (ALS)?
- Have you ever been diagnosed by a doctor with Multiple Sclerosis?
- Have you ever been diagnosed by a doctor with dementia / Alzheimer’s disease?
- Have you ever been diagnosed by a doctor with auto immune disease?

Table I. Correlation table of all variables included in the models (Spearman’s *ρ*).

|  | 1 | 2 | 3 | 4 | 5 | 6 | 7 | 8 | 9 | 10 | 11 | 12 | 13 | 14 |
| --- | --- | --- | --- | --- | --- | --- | --- | --- | --- | --- | --- | --- | --- | --- |
| 1 Base exp 2011 |  |  |  |  |  |  |  |  |  |  |  |  |  |  |
| 2 Electricity exp 2011 | 0.57*** |  |  |  |  |  |  |  |  |  |  |  |  |  |
| 3 Phone exp 2011 | 0.52*** | 0.43*** |  |  |  |  |  |  |  |  |  |  |  |  |
| 4 Road exp 2011 | 0.49*** | 0.41*** | 0.40*** |  |  |  |  |  |  |  |  |  |  |  |
| 5 UV exp 2011 | 0.36*** | 0.29*** | 0.41*** | 0.41*** |  |  |  |  |  |  |  |  |  |  |
| 6 Base exp 2015 | 0.44*** | 0.31*** | 0.32*** | 0.26*** | 0.19*** |  |  |  |  |  |  |  |  |  |
| 7 Electricity exp 2015 | 0.32*** | 0.43*** | 0.25*** | 0.23*** | 0.16*** | 0.56*** |  |  |  |  |  |  |  |  |
| 8 Phone exp 2015 | 0.31*** | 0.23*** | 0.45*** | 0.21*** | 0.23*** | 0.56*** | 0.43*** |  |  |  |  |  |  |  |
| 9 Road exp 2015 | 0.32*** | 0.25*** | 0.27*** | 0.62*** | 0.25*** | 0.39*** | 0.35*** | 0.33*** |  |  |  |  |  |  |
| 10 UV exp 2015 | .19*** | 0.16*** | 0.23*** | 0.19*** | 0.35*** | 0.38*** | 0.28*** | 0.43*** | 0.34*** |  |  |  |  |  |
| 11 Somatisation 2011 | 0.10*** | 0.09*** | 0.09*** | 0.14*** | 0.06*** | 0.08*** | 0.09*** | 0.06*** | 0.12*** | 0.04*** |  |  |  |  |
| 12 Somatisation 2015 | .07*** | 0.08*** | 0.08*** | 0.13*** | 0.05*** | 0.10*** | 0.09*** | 0.09*** | 0.13*** | 0.07*** | 0.67*** |  |  |  |
| 13 Presence of diagnosis | -0.05*** | -0.05*** | -0.03** | -0.01 | -0.02 | -0.05*** | -0.05*** | -0.04*** | -0.00 | -0.01 | 0.22*** | 0.23*** |  |  |
| 14 Age | -0.18*** | -0.17*** | -0.23*** | -0.10*** | -0.19*** | -0.16*** | -0.17*** | -0.24*** | -0.09*** | -0.16*** | 0.02 | 0.02 | 0.27*** |  |
| 15 Sex | 0.05*** | 0.11*** | -0.01 | 0.03* | 0.05*** | 0.06*** | 0.12*** | 0.02 | 0.02 | 0.06*** | 0.17*** | 0.14*** | 0.01 | -0.09*** |

p < .0001 ‘****’; p < .001 ‘***’, p < .01 ‘**’, p < .05 ‘*’

Table II. Descriptives.

|  | mean | sd | min | max | skew | kurtosis | se |
| --- | --- | --- | --- | --- | --- | --- | --- |
| Base 2015 | 1,2 | 1,44 | 0 | 6 | 1,3 | 1,11 | 0,02 |
| Base 2011 | 1,01 | 1,2 | 0 | 6 | 1,46 | 2,2 | 0,01 |
| Electricity 2015 | 0,6 | 0,98 | 0 | 6 | 2,1 | 4,73 | 0,01 |
| Electricity 2011 | 0,51 | 0,82 | 0 | 6 | 2,13 | 5,69 | 0,01 |
| Phone 2015 | 1,91 | 1,5 | 0 | 6 | 0,73 | -0,17 | 0,02 |
| Phone 2011 | 1,66 | 1,25 | 0 | 6 | 0,98 | 0,73 | 0,01 |
| Road 2015 | 1,7 | 1,26 | 0 | 6 | 0,83 | 0,27 | 0,01 |
| Road 2011 | 1,47 | 1,19 | 0 | 6 | 1,01 | 0,69 | 0,01 |
| UV 2015 | 2,08 | 1,51 | 0 | 6 | 0,37 | -0,56 | 0,02 |
| UV 2011 | 1,82 | 1,19 | 0 | 6 | 0,51 | 0,05 | 0,01 |
| Somatisation 2015 | 5,61 | 4,89 | 0 | 32 | 1,35 | 2,14 | 0,06 |
| Somatisation 2011 | 5,62 | 4,98 | 0 | 32 | 1,4 | 2,26 | 0,06 |
| Presence of diagnosis | 0,8 | 0,4 | 0 | 1 | -1,48 | 0,19 | 0 |

Table III. Spline analyses of the different models (NOC E and ATTR E) - (ANOVA tables)

|  | Base | | | Electricity | | | Phone | | | Road | | | UV | | |
| --- | --- | --- | --- | --- | --- | --- | --- | --- | --- | --- | --- | --- | --- | --- | --- |
|  | Chi-Square | d.f. | p | Chi-Square | d.f. | p | Chi-Square | d.f. | p | Chi-Square | d.f. | p | Chi-Square | d.f. | p |
| Baseline Perceived exposure (NOC E) | 0,39 | 1 | 0.53 | 1.46 | 1 | 0.23 | 0.15 | 1 | 0.7 | 2.35 | 1 | 0.12 | 0.36 | 1 | 0.55 |
| Baseline Somatisation (ATTR E) | 0,55 | 1 | 0.46 | 1.05 | 1 | 0.31 | 2.73 | 1 | 0.1 | 3.03 | 1 | 0.08 | 0.33 | 1 | 0.57 |


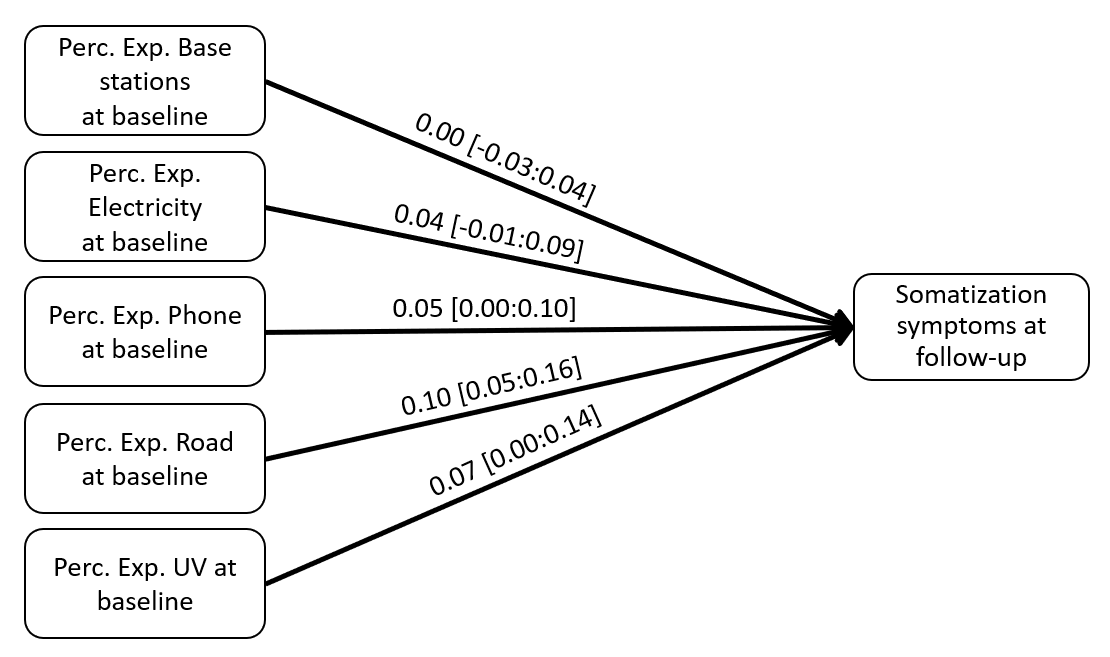


Figure A. Graphical representation of the NOC models, limited to the estimates of the main predictors.


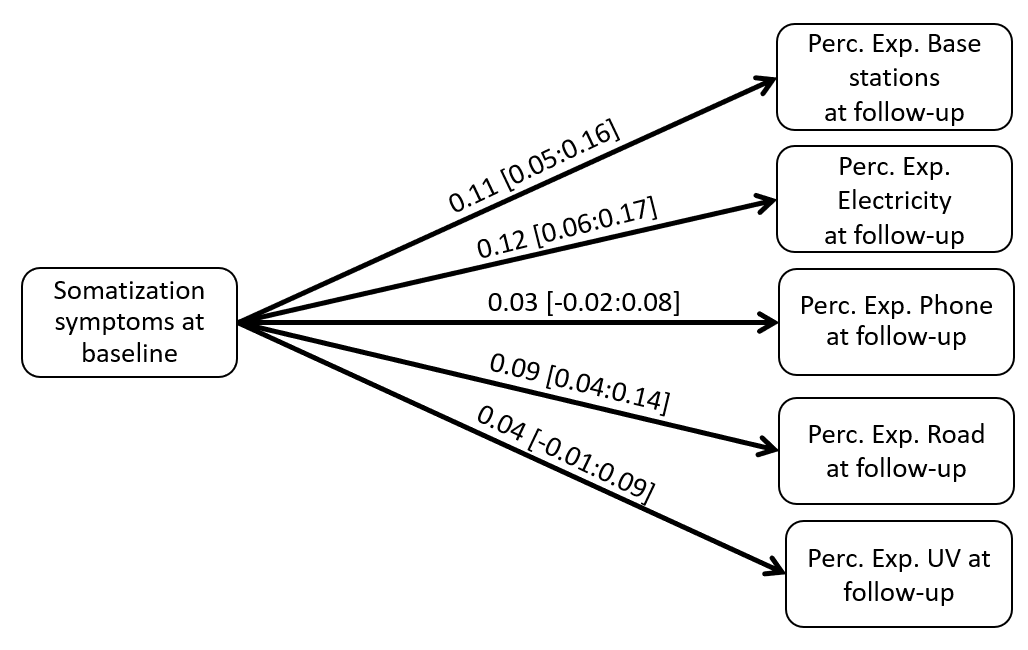


Figure B. Graphical representation of the ATTR models, limited to the estimates of the main predictor.
